# Alterations in serum kynurenine pathway metabolites in individuals with high neocortical amyloid-β load: A pilot study

**DOI:** 10.1038/s41598-018-25968-7

**Published:** 2018-05-22

**Authors:** Pratishtha Chatterjee, Kathryn Goozee, Chai K. Lim, Ian James, Kaikai Shen, Kelly R. Jacobs, Hamid R. Sohrabi, Tejal Shah, Prita R. Asih, Preeti Dave, Candice ManYan, Kevin Taddei, David B. Lovejoy, Roger Chung, Gilles J. Guillemin, Ralph N. Martins

**Affiliations:** 1 Department of Biomedical Sciences, Macquarie University, North Ryde, NSW Australia; 2 School of Medical Health and Sciences, Edith Cowan University, Joondalup, WA Australia; 3 KaRa Institute of Neurological Disease, Sydney, Macquarie Park, NSW Australia; 4 Clinical Research Department, Anglicare, Sydney, Castle Hill, NSW Australia; 5 School of Psychiatry and Clinical Neurosciences, University of Western Australia, Crawley, WA Australia; 6 Australian Alzheimer’s Research Foundation, Nedlands, WA Australia; 7 The Cooperative Research Centre for Mental Health, Carlton South, Vic, Australia; 8 Institute for Immunology & Infectious Diseases, Murdoch University, Murdoch, WA Australia; 9 Australian eHealth Research Centre, CSIRO, Floreat, WA Australia; 10 School of Medical Sciences, University of New South Wales, Kensington, NSW Australia

## Abstract

The kynurenine pathway (KP) is dysregulated in neuroinflammatory diseases including Alzheimer’s disease (AD), however has not been investigated in preclinical AD characterized by high neocortical amyloid-β load (NAL), prior to cognitive impairment. Serum KP metabolites were measured in the cognitively normal KARVIAH cohort. Participants, aged 65–90 y, were categorised into NAL+ (n = 35) and NAL− (n = 65) using a standard uptake value ratio cut-off = 1.35. Employing linear models adjusting for age and *APOE*ε4, higher kynurenine and anthranilic acid (AA) in NAL+ versus NAL− participants were observed in females (kynurenine, p = 0.004; AA, p = 0.001) but not males (NALxGender, p = 0.001, 0.038, respectively). To evaluate the predictive potential of kynurenine or/and AA for NAL+ in females, logistic regressions with NAL+/− as outcome were carried out. After age and *APOE*ε4 adjustment, kynurenine and AA were individually and jointly significant predictors (p = 0.007, 0.005, 0.0004, respectively). Areas under the receiver operating characteristic curves were 0.794 using age and *APOE*ε4 as predictors, and 0.844, 0.866 and 0.871 when kynurenine, AA and both were added. Findings from the current study exhibit increased KP activation in NAL+ females and highlight the predictive potential of KP metabolites, AA and kynurenine, for NAL+. Additionally, the current study also provides insight into he influence of gender in AD pathogenesis.

## Introduction

**Figure 1:**
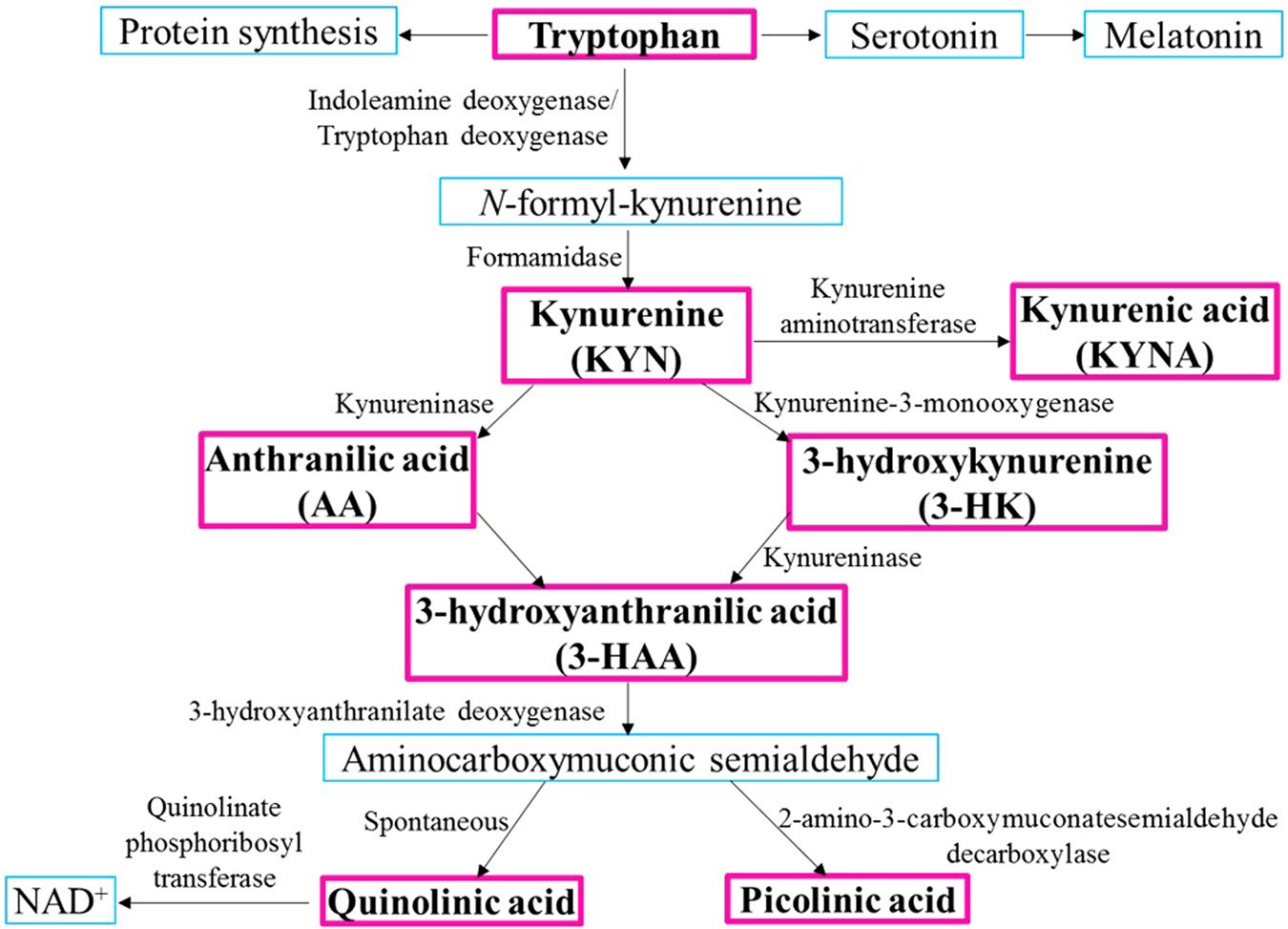
Schematic diagram of the kynurenine pathway. Tryptophan can be utilised for protein synthesis or serotonin and melatonin production. However, over 90% peripheral tryptophan has been reported to be metabolised via the kynurenine pathway (KP) in mammals. In the KP, tryptophan is metabolised into *N*-formyl-kynurenine followed by kynurenine (KYN) catalysed by enzymes indoleamine deoxygenase or tryptophan deoxygenase and formamidase. KYN is then converted to kynurenic acid (KYNA) by enzyme kynurenine aminotransferase. Alternatively, KYN is metabolised to anthranilic acid (AA) and 3-hydroxykynurenine (3-HK) by enzymes kynureninase and kynurenine-3-monooxygenase, respectively. AA and 3-HK further get metabolised to 3-hydroxyanthranilic acid (3-HAA), which in turn gets converted to aminocarboxymuconic semialdehyde (ACMA). ACMA spontaneously converts to neurotoxin, quinolinic acid, a substrate for the redox agent, NAD+. Alternatively, ACMA can be assisted by enzyme 2-amino-3-carboxymuconatesemialdehyde decarboxylase to generate picolinic acid. KP metabolites marked in bold, have been investigated in the current study.

The main physiological roles of tryptophan metabolism are to generate serotonin and melatonin and also, the essential co-factor nicotinamide adenine dinucleotide (NAD^+^) through the kynurenine pathway (KP) (Fig. 1). In neuroinflammatory conditions, the KP is strongly up regulated leading to the production of several neuroactive metabolites that can be either neuroprotective, neurotoxic or immuno-modulatory. It has previously been demonstrated that the KP is activated in several neurodegenerative and neuropsychiatric disorders including Alzheimer’s disease (AD)^1–6^.

Interestingly, this central dysregulation of the KP homeostasis also manifests in the blood in AD patients^7–9^. Higher ratios of KP metabolites, kynurenine (KYN) to tryptophan (K:T) in serum and plasma have been reported in patients with AD and mild cognitive impairment (MCI)^10,11^ and this ratio (K:T) also inversely correlated with cognitive performance^10^. Further, a decline in plasma and erythrocyte concentrations of the KP metabolite kynurenic acid (KYNA), which is produced via a secondary branch of the KP and precludes NAD^+^ production from KYN, has been reported in patients with AD and MCI^11,12^. Furthermore, elevated plasma levels of the excitotoxin quinolinic acid, have been reported in AD^7^. Additionally, a relatively recent study reported an association between dementia risk and elevated plasma levels of the KP metabolite, anthranilic acid (AA)^13^.

However, KP metabolite alterations have never been investigated in the preclinical stage of AD that is characterised by high neocortical amyloid-β load (NAL)^14^ measured via positron emission tomography (PET), prior to cognitive decline, given that the deposition of NAL begins to occur two to three decades prior to the clinical manifestation of the disease^15^.

Therefore, the current pilot study investigated whether the dysregulation of the KP occurs within the preclinical stage of the AD pathogenesis trajectory, in cognitively normal individuals. Serum tryptophan and KP metabolites, primarily comprising, KYN, KYNA, 3-hydroxykynurenine (3-HK), 3-hydroxyanthranilic acid (3-HAA), AA, picolinic acid and quinolinic acid, were hence measured in, and compared between, cognitively normal individuals with preclinical AD characterised by high NAL (NAL+; standard uptake value ratio (SUVR) ≥1.35) and individuals with no apparent risk to AD, characterised by low NAL (NAL−, SUVR <1.35)^16–18^.

## Results

### Comparison of demographic characteristics between NAL− and NAL+ participants

Demographic characteristics of study participants have been presented in Table 1 (and demographic characteristics stratified by gender are available in Supplementary Table 1). As expected, the *APOE* ε4 carriage frequency was significantly higher in NAL+ participants (45.7%) compared to NAL− participants (7.7%)^18–20^. No significant differences were observed in age, gender, body mass index, MMSE scores, MoCA scores, years of education, sex hormone levels and hippocampal volume between NAL− and NAL+ cohort participants (Table 1).

**Table 1:** Demographic characteristics of cohort participants.

	NAL−	NAL+	p
Gender (M/F)	19/46	13/22	0.419
Age (years, mean ± SD)	77.61 ± 5.55	79.22 ± 5.38	0.165
BMI (mean ± SD)	27.38 ± 4.47	28.05 ± 4.73	0.486
n*APOE ε4* carriers (%)	5 (7.7)	16 (45.7)	<0.0001
MMSE (mean ± SD)	28.50 ± 1.16	28.80 ± 1.10	0.225
MoCA (mean ± SD)	27.43 ± 1.67	27.03 ± 1.92	0.278
Education (years, mean ± SD)	14.84 ± 3.37	13.64 ± 2.91	0.078
Testosterone (nmol/L, mean ± SD), males	14.43 ± 6.16	11.66 ± 3.77	0.160
Testosterone (nmol/L, mean ± SD), females	1.45 ± 2.55	1.26 ± 0.81	0.738
Oestradiol (pmol/L, mean ± SD), males	113.16 ± 47.83	117.23 ± 35.17	0.795
Oestradiol (pmol/L, mean ± SD), females	91.22 ± 118.71	73.90 ± 28.97	0.523
FBB-PET SUVR (mean ± SD, n = 100)	1.15 ± 0.08	1.71 ± 0.26	—
HV% (left; right lobes, mean ± SD, n = 96)	0.195 ± 0.020; 0.199 ± 0.021	0.194 ± 0.019; 0.199 ± 0.018	0.805; 0.890

Baseline characteristics including gender, age, body mass index (BMI), *APOE ε4* status, mini mental state examination (MMSE) scores, Montreal Cognitive Assessment (MoCA, adjusted for education) scores, years of education, sex hormone levels, neocortical amyloid load (NAL) represented by the standard uptake value ratio (SUVR) of ligand ^18^F-Florbetaben (FBB) in the neocortical region normalised with that in the cerebellum, and hippocampal volume (HV) normalised by the intracranial volume, have been compared between NAL− (SUVR <1.35) and NAL+ (SUVR ≥1.35) study participants. Chi-square test or linear models were employed as appropriate. Ninety-six participants underwent MRI (nNAL− = 64, nNAL+ = 32).

### KP metabolites and the three major AD risk factors i.e. age, APOE ε4 carriage and gender

When the association between KP metabolites and AD risk factors were investigated, among the KP metabolites, quinolinic acid positively correlated with age (p < 0.05, Supplementary Table 2), while no significant differences in KP metabolites were observed with respect to *APOE* ε4 carriage (Supplementary Table 3). However, significantly higher concentrations of KP metabolites, KYNA and 3-HAA, were observed in males compared to females, uncorrected and corrected for covariates age and *APOE* ε4 status (p ≤ 0.005, Supplementary Table 4).

### KP metabolites and NAL

**Figure 2:**
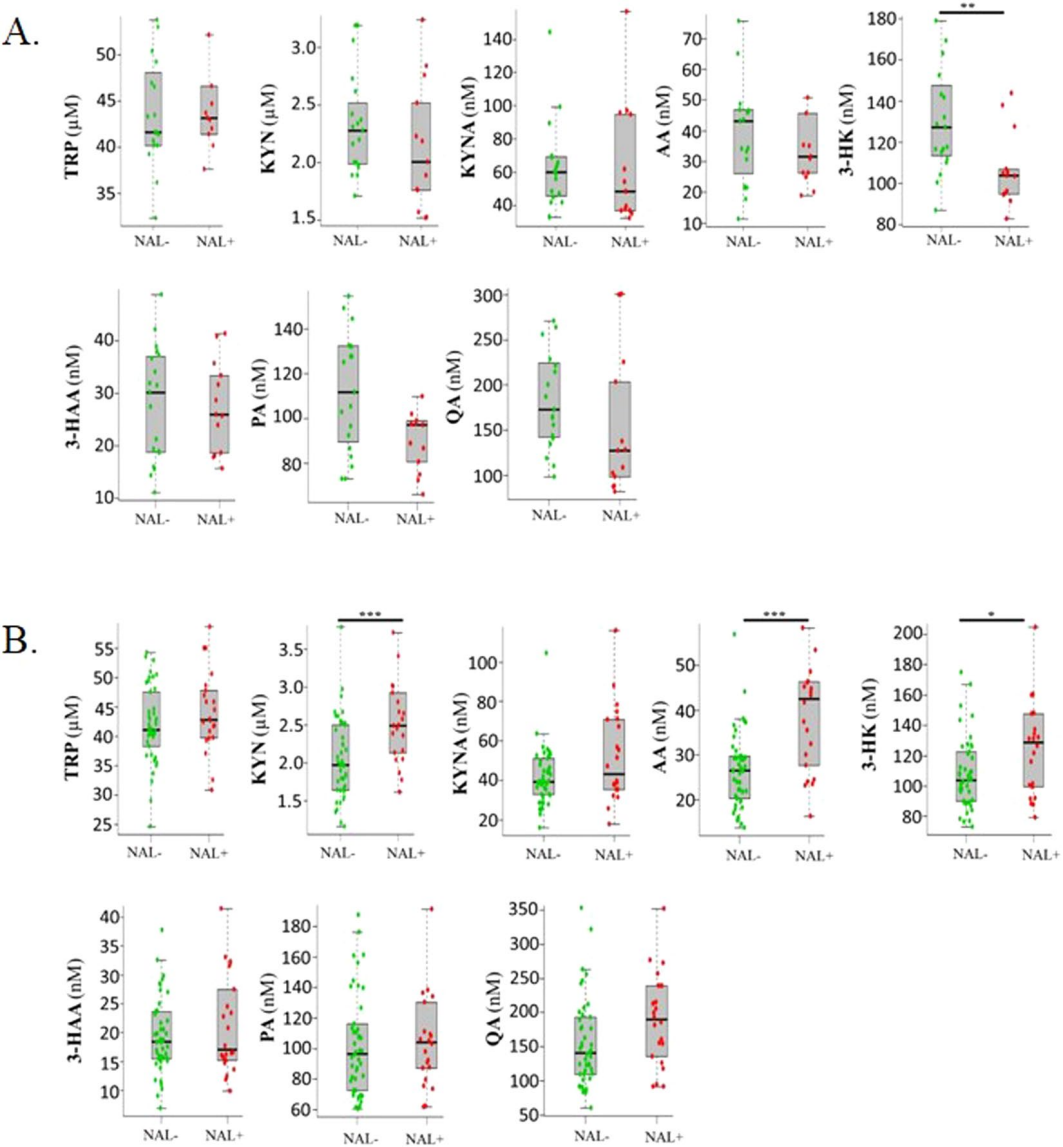
Comparison of kynurenine pathway metabolites between NAL− and NAL+ participants, separately in males and females. Kynurenine pathway metabolite concentrations in serum were compared between participants (in **A**. Males and **B**. Females, separately) with neocortical amyloid-β load (NAL, assessed by the standard uptake value ratio observed via positron emission tomography using ligand ^18^F-florbetaben) <1.35 (NAL−) and ≥1.35 (NAL+) using linear models. Kynurenine (KYN), anthranilic acid (AA) and 3-hydroxykynurenine (3-HK) levels were significantly higher in NAL+ (N = 22) compared with NAL− (N = 46) female participants while 3-hydroxykynurenine (3-HK) levels were significantly lower in NAL+ (N = 13) compared with NAL− (N = 19) male participants. The line segment within each box plot represents the median within each box plot and error bars represent the range of the metabolite concentration for each group. *Represents p < 0.05, **represents p ≤ 0.01 and ***represents p < 0.0005; p values were obtained from variables transformed to the logarithmic scale for analyses to meet assumptions of the statistical test employed. TRP, tryptophan; KYNA, kynurenic acid; HAA, 3-hydroxyanthranilic acid; PA, picolinic acid; QA, quinolinic acid. (**A**) Serum metabolite concentrations in males. (**B**) Serum metabolite concentrations in females.

Serum kynurenine and anthranilic acid levels were significantly elevated in NAL+ versus NAL− participants uncorrected and corrected for age, gender, *APOE* ε4 status and education (Supplementary Table 5). However, on comparing KP metabolites between NAL− and NAL+ cohort participants, after adjustment for age, gender and *APOE* ε4 status, there were significant differences between the associations of metabolites and NAL+/− according to gender for KYN (p = 0.001), 3- HK (p = 0.001), AA (p = 0.038) and QA (p = 0.007). In particular, KYN was significantly higher for NAL+ vs NAL− in females (p = 0.004), but not significantly different in males; 3-HK was higher for NAL+ vs NAL− in females (p = 0.017) but lower in males (p = 0.022); and AA was higher for NAL+ vs NAL− in females (p = 0.001) but not for males. QA showed a tendency to increase for NAL+ vs NAL− in females and to decrease in males, but neither were individually significant (Fig. 2, Supplementary Table 6, demographics available in Supplementary Table 1). Additionally, given the complex interaction between KP metabolites and that the generation of KYNA precludes the formation of AA given their common precursor (KYN, see Fig. 1), we also investigated K:T and AA:KYNA ratios, wherein both were observed to be significantly higher in NAL+ versus NAL− female participants (Supplementary Table 6).

**Figure 3:**
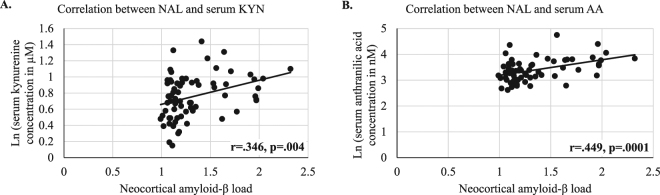
Correlation between NAL and KP metabolites, KYN and AA, in females. Significant correlations were observed between neocortical amyloid-β load (NAL) and serum concentrations of **(A)** kynurenine (KYN; r = 0.346, p = 0.004, n = 68) **(B)** anthranilic acid (AA; r = 0.449, p = 0.0001, n = 68) using Pearson’s correlation coefficient (r), in females. Correlation coefficients after adjusting for covariates age and *APOE*ε4 are available in Supplementary Table 6. Serum KYN and AA concentrations were transformed to the logarithmic scale for analyses, to meet assumptions of the statistical test employed.

While no correlation between NAL (as a continuous variable) and KP metabolites were observed in male participants, NAL significantly correlated with KYN and AA without adjusting for covariates (Fig. 3) and KYN, AA, 3-HK and 3-HAA after correcting for covariates age and *APOE* ε4 status in female participants (Supplementary Table 7). The observation of 3-HAA significantly correlating with NAL in the females, but not being significantly elevated in NAL+ versus NAL− for this gender (Supplementary Table 6) can be attributed to the fact that 3-HAA correlated with NAL (correcting for age and *APOE* ε4 status) only within the NAL+ female subset (n = 22, r^a^ = 0.572, p^a^ = 0.008).

### Potential of KP metabolites to predict NAL+

**Figure 4:**
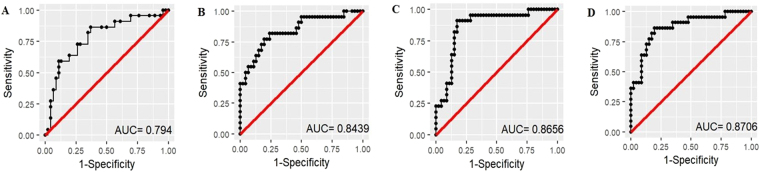
Receiver operating characteristic (ROC) curves for the prediction of high neocortical amyloid-β load in female participants. The ‘base’ model comprising major risk factors age and *APOE* ε4 allele status (**A**) was outperformed by the ‘base+ kynurenine’ model (**B**) and the ‘base+ anthranilic acid’ model (**C**) and ‘base+ kynurenine+ anthranilic acid’ model (**D**). Logistic regression models were employed to perform the analyses. AUC: area under the curve.

Given that significantly elevated KYN and AA concentrations, in particular, were observed in NAL+ (versus NAL−) female sera we evaluated these and other metabolites as potential markers in females to predict NAL+ via logistic regression with NAL+/− as response. After adjusting for age and *APOE* ε4 status, all metabolites were entered jointly (on the log scale) and backward elimination was used to obtain the simplest model containing only significant terms. Only KYN (p = 0.03) and AA (p = 0.045) remained jointly significant (joint p = 0.0004). A ‘base’ model incorporating the major risk factors for AD, age and *APOE* ε4 allele status, was then generated and compared to the ‘base+ KYN’, ‘base+ AA’ and ‘base+ KYN+ AA’ models, wherein either KYN, AA or both KYN and AA concentrations were added to the base model (Fig. 4). The area under the curve (AUC) of the ‘base+ KYN’ (AUC = 0.843, specificity = 52% at sensitivity = 90%, log(KYN) p = 0.007), ‘base+ AA’ (AUC = 0.865, specificity = 83% at sensitivity = 90%, log(AA) p = 0.005) and ‘base+ KYN+ AA’ model (AUC = 0.87, specificity = 65% at sensitivity = 90%) outperformed the ‘base’ model (AUC = 0.794, specificity = 43% at sensitivity = 90%) in distinguishing NAL+ from NAL− participants. While it is marginally significant on its own as an additional predictor for NAL+, the K:T ratio is not significant when AA is already included (p = 0.47).

## Discussion

In mammals, the KP has been reported to account for over 90% of peripheral tryptophan catabolism^21^. Our findings exhibit aberrant peripheral tryptophan metabolism, via the KP, in preclinical AD wherein, significantly elevated KYN, AA and 3-HK serum concentrations were present in NAL+ versus NAL− females. Additionally, within female study participants, a significant positive correlation was observed between NAL and the aforementioned serum KP metabolite concentrations.

Similar to findings in the current preclinical AD study, elevated serum KYN concentrations and K:T ratios have been reported previously in clinical AD and in individuals with MCI^10,11^. The foremost step in the KP i.e. the generation of KYN from tryptophan, via *N*-formyl-KYN, is catalysed by enzymes indoleamine 2,3-dioxygenase 1(IDO1; predominantly expressed in extra-hepatic cells such as neurons, macrophages, microglia and astrocytes) or tryptophan 2,3-dioxygenase (TDO; predominantly expressed in hepatocytes)^22–25^. Significantly higher IDO1 and TDO immunoreactivity has been reported in AD patient hippocampi^3,26^. Further, IDO1 and TDO, have been reported to be regulated by immune system signalling molecules, growth factors and steroids^1,22,27,28^, all of which have been shown to be associated with AD pathogenesis^29–33^. Our findings of elevated serum KYN concentrations along with higher K:T ratios in NAL+ versus NAL− participants together with the afore cited literature are therefore indicative of increased tryptophan degradation via the KP, from the preclinical stages of the AD pathogenesis trajectory, prior to cognitive impairment.

Given the observation of significantly increased KP activation in NAL+ participants, apparent from the elevated serum KYN concentrations and K:T ratios in NAL+ (female) participants within the current study, further investigation of downstream KP metabolites revealed elevated AA serum concentrations in females. This observation is further corroborated by a study conducted by Chouraki and colleagues that reported elevated serum AA concentrations in cognitively normal individuals who later developed dementia^13^. However, AA concentrations were not reported to be significantly elevated in a study examining KP metabolites in clinical AD^7^. Taken together with the findings of Chouraki and colleagues, we specifically demonstrate elevated AA levels as a feature of NAL+ preclinical AD that is predictive of converting to clinical AD. However, our study shows a strong gender effect, namely in females.

Additionally, it is important to note that even though both precursors to 3-HAA (AA, 3-HK) are elevated in NAL+ females, 3-HAA itself is not elevated in NAL+ females. Interestingly, Chouraki and colleagues reported a trend of association between plasma 3-HK and decreased risk of incident dementia, even though plasma anthranilic acid levels were observed to be associated with increased risk of incident dementia^13^. One possible explanation could be that the enzyme (activity) that catalyses the production of 3-HAA is decreased in NAL+, however, further studies are required to investigate this notion.

While the influence of gender on serum KP metabolite concentrations has been reported previously^34^, to our knowledge, no previous study has reported significantly elevated KYN (or K:T ratio) or/and AA or/and 3-HK restricted to the female population, in AD pathogenesis, as observed within the current study. It is however acknowledged that the current cohort may have lacked sufficient power within the male subset given its modest sample size (19 NAL− males/13 NAL+ males). Three-HK was observed to significantly increase in NAL+ females compared to NAL− females but decrease in NAL+ males compared to NAL− males. While the mechanism behind these observations are currently not known, our results further exhibit the strong gender difference in AD pathogenesis (Supplementary Table 6). Schwarz and colleagues reported elevated 3-HK serum concentrations in AD, which support our findings in preclinical female participants. Interestingly, the clinical AD group in the study by Schwarz and colleagues comprised 80% females (16 females/4 males)^8^. Given the paucity of literature describing the impact of gender on the relationship between AD pathology and 3-HK and particularly the relatively small sample size of males in both studies, further investigations are warranted on KP metabolite alterations in AD pathogenesis, in larger male and female cohorts.

Further, the current study observed a strong interaction effect between NAL and gender on KP metabolites that may be attributed to the influence of sex hormones on the enzymes involved within the KP^35^. For example, testosterone has been reported to have an inhibitory effect on the KP (via TDO/IDO1 by reducing NAD production in favour of KYNA formation)^36,37^ while oestrogen has been reported to induce (via TDO/IDO1 favouring NAD^+^ production, precluding KYNA formation)^38^ as well as inhibit the KP (by inhibiting immune system activation)^39^. However, the incidence of ageing-related hormonal changes prevalent within the KARVIAH cohort and the complex interplay and feedback mechanisms between the various KP metabolites also need to be considered with regard to observations in this study. Within the current study, it may be posited that elderly women were more vulnerable (NAL+ vs NAL−) to increased KP activity compared to elderly men, given the postmenopausal plummet in oestrogen which increases vulnerability to AD, while in similarly aged men^40^, testosterone levels are relatively stable which may confer neuroprotection^32,33,41–45^.

In addition to the influence of the sex hormones, other mechanisms resulting in increased KP activation contributing to AD pathogenesis may also be involved. Given that inflammation is an early feature in the AD pathogenesis trajectory, inflammation induced immune responses occurring in AD pathogenesis, may upregulate IDO activity and tryptophan degradation resulting in increased KP activation^46,47^. Although the current study addresses the peripheral concentrations of KP metabolites, blood concentrations of tryptophan, KYN, AA and 3-HK significantly contribute to the brain pools of these metabolites (~60%), given their permeability to the blood brain barrier^48,49^. Therefore, elevated KYN, AA and 3-HK in NAL+ serum may consequently enhance KYN, AA and 3-HK concentrations in NAL+ brains. Given that the two branches of the KP occur in a segregated manner in the brain, wherein 3-HK and its further downstream metabolites occur in the microglia (while KYNA is formed in the astrocytes)^25^, we posit that an increase in KP intermediate substrates impact microglial activation inducing secretion of inflammatory signalling molecules. This inflammation could further induce Aβ generation, in turn triggering further inflammation, resulting in a vicious cycle^50^. Aβ has been reported to induce interferon-γ^51^, a potent activator of IDO^52^, resulting in increased KP activity^53^, further leading to elevated concentrations of KYN and its downstream metabolites, such as AA. It could be argued that elevations in AA concentrations in NAL+ versus NAL− may actually represent a protective mechanism against AD pathogenesis due to the presence of iron dyshomeostasis in preclinical AD^17,54^, given that AA is known to have reactive oxygen species scavenging properties by iron chelation^55^.

The current pre-mortem gold standard biomarkers for preclinical AD, namely NAL measured via PET (employed within the current study) and cerebrospinal fluid (CSF) concentrations of Aβ42 and tau, are uneconomical and invasive, respectively. Therefore, we also evaluated the KP metabolite alterations observed within the present study as potential blood biomarkers for preclinical AD or NAL+ . We found significant KP metabolite alterations that reflect NAL positivity in females. Interestingly, the addition of AA to a base model comprising age and *APOE* ε4 allele status, resulted in an increased specificity from 43% to 83%, at a sensitivity of 90%, indicating that AA may serve as a statistically significant predictor of NAL over and above the base model, in elderly females.

We acknowledge that the current study has limitations such as the cross-sectional nature of the study and the relatively modest sample size of the cohort whereby the male subset may have lacked sufficient power, contributing to the absence of significant KP metabolite alterations observed in the female cohort subset. However, given that sex hormones play an important role in regulating the KP, it is highly likely that the findings in our study will be replicated in a larger cohort. Additionally, given the modest sample size and pilot nature of the study, we have not corrected for multiple testing. However, our findings of significantly elevated KYN and AA levels in NAL+ females compared to NAL− females remain significant following Bonferroni correction for multiple testing (KYN, p = 0.03; AA, p = 0.01). Furthermore, although KYN and AA significantly add to the predictive potential of the base model comprising the three major risk factors of AD (namely age, *APOE* ε4 and gender) for NAL+, further confirmatory studies are required.

Finally, to conclude, the current study reports KP metabolite alterations in preclinical AD (characterised by high NAL) females wherein, serum concentrations of KYN, AA and 3-HK were elevated prior to cognitive impairment and hippocampal atrophy. To our knowledge, this is also the first study to report an association between concentrations of the aforementioned metabolites with NAL, a gold standard biomarker of AD. Importantly, the current study also identifies potential blood biomarkers for preclinical AD. Interestingly, our findings were restricted to the female subset of the cohort, which may provide further insight into the gender effects in AD pathogenesis. While our observations could be attributed to the several mechanisms hypothesised above, further validation studies are warranted. Given the preliminary nature of the current findings, confirmation in a larger sample set is required. Additionally, future studies are also required to measure the KP enzyme activities, in addition to employing male predominant cohorts and longitudinal studies, to gain further insight into these current findings.

## Methods and Materials

### Participants

Study participants were from the Kerr Anglican Retirement Village Initiative in Ageing Health (KARVIAH) cohort, at baseline. All participants were residents of Anglicare, New South Wales, Australia.

Cohort volunteers (N = 206) were screened for the inclusion and exclusion criteria of the KARVIAH cohort^17^. The inclusion criteria for the KARVIAH Study briefly comprised, an age range of 65–90 years, good general health, no known significant cerebral vascular disease, fluent in English, adequate/corrected vision and hearing to enable testing, no objective cognitive impairment measured using a Montreal Cognitive Assessment (MoCA) cut-off score ≥26^56^. MoCA scores lying between 18–25 were assessed on a case by case basis by the study neuropsychologist following stratification of scores according to age and education norms^57^. The exclusion criteria comprised, the diagnosis of dementia based on the revised criteria from the National Institute on Aging - Alzheimer’s Association^58^, presence of acute functional psychiatric disorder (including lifetime history of schizophrenia or bipolar disorder), history of stroke, severe or extremely severe depression (based on the depression, anxiety, stress scales; DASS) and uncontrolled hypertension (systolic BP > 170 or diastolic BP > 100).

One hundred and five participants out of the 134 volunteers meeting the inclusion/exclusion criteria, underwent neuroimaging, neuropsychometric evaluation and blood collection, as the remaining either declined neuroimaging or withdrew from the study. Within these 105 participants, 100 participants were considered to be cognitively normal based on their Mini-Mental State Examination score^59^ (MMSE ≥26), and were included in the current study. Concentrations of the KP metabolites in the serum were reported in all 100 participants with MMSE ≥26. All experimental protocols were approved by the Bellberry Human Research Ethics Committee, Australia and were performed in accordance with the relevant guidelines and regulations. The study volunteers provided written informed consent prior to participation, and the Bellberry Human Research Ethics Committee, Australia, provided ethical approval for the study.

### Evaluation of neocortical amyloid-β load and hippocampal volumes via PET and MRI

Study participants were imaged within three months of blood collection. Participants underwent positron emission tomography (PET) using ligand ^18^F-Florbetaben (FBB), and magnetic resonance imaging (MRI) at Macquarie Medical Imaging in Sydney.

All 100 participants within the current study underwent FBB-PET. Participants were administered an intravenous bolus of FBB slowly over 30 s, while in a rested position. Images were acquired over a 20 min scan, in 5 min acquisitions, beginning 50 min post injection. Neocortical amyloid load was calculated as the mean SUVR of the ligand FBB in the frontal, superior parietal, lateral temporal, lateral occipital, and anterior and posterior cingulate regions normalised with that in the cerebellum using image processing software, CapAIBL^60,61^.

Ninety-six of the 100 participants within the current study passed all standard MRI inclusion/exclusion criteria, and underwent MRI as described previously using a General Electric (GE) 3Tesla scanner (Model 750 W)^16^. Hippocampal volume calculated from the images acquired was normalized with the total intracranial volume comprising the cerebrospinal fluid, grey matter and white matter.

### Blood collection and processing, and measurement of KP metabolites in serum

All study participants fasted for a minimum of 10 hours overnight prior to blood collection employing standard serological methods and processing techniques for serum collection as described previously^17,20^. Serum samples were collected in the year 2015 and stored at −80 °C until thawed for measurement of KP metabolites in the year 2017.

All reagents and KP metabolite standards were analytical reagent grade and were purchased from Sigma-Aldrich (St Louis, MO), unless otherwise stated. Deuterated internal standards were purchased from Medical Isotopes, Inc (Pelham, NH). Serum samples were prepared for KP metabolite analyses with the addition of equal volumes of 10% (w/v) trichloroacetic acid (TCA).

Analyses of tryptophan, KYN, 3-HK, 3-HAA and AA were performed simultaneously with ultra high performance liquid chromatography (UHPLC) with an injection volume of 20 μL per sample, as described previously^46^. KYNA separation was carried out following injection (10 µL) onto a Poroshell RRHT C-18, 1.8μm 2.1 × 100 mm column (Agilent Technologies, Inc, Santa Clara, CA) and quantified via fluorescence detection (excitation and emission wavelengths of 344 nm and 388 nm, respectively; retention time 1.5 min). A gradient method with 50 mM sodium acetate, 25 mM zinc acetate and 2.25% acetonitrile (Eluent A), and 10% acetonitrile (Eluent B) was employed and separation was performed at 38 °C with a flow rate of 0.75 mL/min. Separation achieved over 10 min, comprised 100% eluent A for 3 min, 50% eluent A for 2 min, 0% eluent A for 2 min and re-equilibration with 100% eluent A for 3 min. Data were analysed with the Agilent OpenLAB CDS ChemStation (Edition C.01.04).

For gas chromatography coupled with mass spectrometry (GCMS), 50 μL of sample extract was derivatized. Simultaneous analysis of picolinic acid and quinolinic acid were performed as described previously^62^ with minor modifications using an Agilent 7890 A GC system coupled with Agilent 5975 C mass spectrometry detector and Agilent 7693 A autosampler (Agilent Technologies, Inc, Santa Clara, CA) with one microliter of derivatized mixture. Separation of picolinic acid and quinolinic acid were achieved with a DB-5MS column, 0.25μm film thickness, 0.25 mm × 30 m capillary column (Agilent Technologies, Inc, Santa Clara, CA) within 7 min but the assay run time was set for 12 min to prevent sample carryover. Picolinic acid and quinolinic acid concentrations were analysed using Agilent GC/MSD ChemStation software (Edition 02.02.1431) and interpolated from the established six-point calibration curves based on the abundance count ratio of the metabolites to their corresponding deuterated internal standards within each standard and sample. The intra- and inter-assay CV was within the acceptable range (4–8% for UHPLC assays, 7–10% for GCMS assays) calculated from the repeated measures of the metabolite standards incorporated during the sequence run.

### APOE genotyping

Apolipoprotein E (*APOE)* genotype was determined from purified genomic DNA extracted from 0.5 ml whole blood, wherein each sample was genotyped for the presence of the ε2, ε3, and ε4 *APOE* variants based on TaqMan SNP genotyping assays for rs7412 (C 904973) and rs429358 (C 3084793) as per the manufacturer’s instructions (AB Applied Biosystems by Life Technologies, Victoria, Australia).

### Statistical analyses

Descriptive statistics including means and standard deviations or proportions were calculated for NAL+ and NAL− groups, with comparisons employing t-tests or Chi-square tests as appropriate in Table 1. Linear models were employed to compare continuous variables (KP metabolites) between categories corrected for covariates age, gender, *APOE* ε4 carrier status and years of education. Response variables were log transformed as necessary to better approximate normality and variance homogeneity. Differences in association of the KP metabolites with NAL+/− for females and males were assessed by incorporation of gender by NAL interactions into the models. Pearson correlations (r) and partial correlations (r^a^, adjusting for age and *APOE* ε4 carrier status and r^b^, adjusting for age, *APOE* ε4 carrier status and education) were employed to evaluate the strength of the association between KP metabolites and neocortical Aβ deposition. Logistic regression with NAL−/+ as response was used to evaluate predictive models and receiver operating characteristic curves constructed from the logistic scores. All analyses were carried out using IBM^®^ SPSS^®^ Version 23 and receiver operating characteristic (ROC) curves were generated using the package Deducer on R (version 3.2.5). The datasets generated and analysed during the current study are available from the corresponding author on reasonable request.

## Electronic supplementary material

Supplementary Information

## References

[1] Schwarcz R, Bruno JP, Muchowski PJ, Wu HQ (2012). Kynurenines in the mammalian brain: when physiology meets pathology. Nature reviews. Neuroscience.

[2] Bonda DJ (2010). Indoleamine 2,3-dioxygenase and 3-hydroxykynurenine modifications are found in the neuropathology of Alzheimer’s disease. Redox report: communications in free radical research.

[3] Guillemin GJ, Brew BJ, Noonan CE, Takikawa O, Cullen KM (2005). Indoleamine 2,3 dioxygenase and quinolinic acid immunoreactivity in Alzheimer’s disease hippocampus. Neuropathology and applied neurobiology.

[4] Dezsi L, Tuka B, Martos D, Vecsei L (2015). Alzheimer’s disease, astrocytes and kynurenines. Curr Alzheimer Res.

[5] Oxenkrug, G., van der Hart, M., Roeser, J. & Summergrad, P. Peripheral Tryptophan - Kynurenine Metabolism Associated with Metabolic Syndrome is Different in Parkinson’s and Alzheimer’s Diseases. *Endocrinol Diabetes Metab J* **1** (2017). PMC5747375 29292800

[6] Trushina E, Dutta T, Persson XM, Mielke MM, Petersen RC (2013). Identification of altered metabolic pathways in plasma and CSF in mild cognitive impairment and Alzheimer’s disease using metabolomics. PLoS One.

[7] Gulaj E, Pawlak K, Bien B, Pawlak D (2010). Kynurenine and its metabolites in Alzheimer’s disease patients. Adv Med Sci.

[8] Schwarz MJ, Guillemin GJ, Teipel SJ, Buerger K, Hampel H (2013). Increased 3-hydroxykynurenine serum concentrations differentiate Alzheimer’s disease patients from controls. Eur Arch Psychiatry Clin Neurosci.

[9] Giil, L. M. *et al*. Kynurenine Pathway Metabolites in Alzheimer’s Disease. *J Alzheimers Dis*, 10.3233/JAD-170485 (2017). 10.3233/JAD-170485 28869479

[10] Widner B (2000). Tryptophan degradation and immune activation in Alzheimer’s disease. J Neural Transm (Vienna).

[11] Greilberger J (2010). Carbonyl proteins as a clinical marker in Alzheimer’s disease and its relation to tryptophan degradation and immune activation. Clin Lab.

[12] Hartai Z (2007). Decreased serum and red blood cell kynurenic acid levels in Alzheimer’s disease. Neurochem Int.

[13] Chouraki, V. *et al*. Association of amine biomarkers with incident dementia and Alzheimer’s disease in the Framingham Study. *Alzheimers Dement*, 10.1016/j.jalz.2017.04.009 (2017). 10.1016/j.jalz.2017.04.009 PMC5722716 28602601

[14] Dubois B (2016). Preclinical Alzheimer’s disease: Definition, natural history, and diagnostic criteria. Alzheimers Dement.

[15] Villemagne VL (2013). Amyloid beta deposition, neurodegeneration, and cognitive decline in sporadic Alzheimer’s disease: a prospective cohort study. Lancet Neurol.

[16] Goozee K (2017). Alterations in erythrocyte fatty acid composition in preclinical Alzheimer’s disease. Sci Rep.

[17] Goozee, K. *et al*. Elevated plasma ferritin in elderly individuals with high neocortical amyloid-beta load. *Mol Psychiatry*, 10.1038/mp.2017.146 (2017). 10.1038/mp.2017.146 28696433

[18] Burnham SC (2014). A blood-based predictor for neocortical Abeta burden in Alzheimer’s disease: results from the AIBL study. Mol Psychiatry.

[19] Mattsson N, Andreasson U, Zetterberg H, Blennow K (2017). & Alzheimer’s Disease Neuroimaging, I. Association of Plasma Neurofilament Light With Neurodegeneration in Patients With Alzheimer Disease. JAMA Neurol.

[20] Ellis KA (2009). The Australian Imaging, Biomarkers and Lifestyle (AIBL) study of aging: methodology and baseline characteristics of 1112 individuals recruited for a longitudinal study of Alzheimer’s disease. Int Psychogeriatr.

[21] Leklem JE (1971). Quantitative aspects of tryptophan metabolism in humans and other species: a review. Am J Clin Nutr.

[22] Salter M, Pogson CI (1985). The role of tryptophan 2,3-dioxygenase in the hormonal control of tryptophan metabolism in isolated rat liver cells. Effects of glucocorticoids and experimental diabetes. Biochem J.

[23] Miller CL (2004). Expression of the kynurenine pathway enzyme tryptophan 2,3-dioxygenase is increased in the frontal cortex of individuals with schizophrenia. Neurobiol Dis.

[24] Guillemin GJ, Smythe G, Takikawa O, Brew BJ (2005). Expression of indoleamine 2,3-dioxygenase and production of quinolinic acid by human microglia, astrocytes, and neurons. Glia.

[25] Guillemin GJ (2001). Kynurenine pathway metabolism in human astrocytes: a paradox for neuronal protection. J Neurochem.

[26] Wu W (2013). Expression of tryptophan 2,3-dioxygenase and production of kynurenine pathway metabolites in triple transgenic mice and human Alzheimer’s disease brain. PLoS One.

[27] Espey MG, Namboodiri MA (2000). Selective metabolism of kynurenine in the spleen in the absence of indoleamine 2,3-dioxygenase induction. Immunol Lett.

[28] Huang L, Baban B, Johnson BA, Mellor AL (2010). Dendritic cells, indoleamine 2,3 dioxygenase and acquired immune privilege. Int Rev Immunol.

[29] Ray S (2007). Classification and prediction of clinical Alzheimer’s diagnosis based on plasma signaling proteins. Nat Med.

[30] Doecke JD (2012). Blood-based protein biomarkers for diagnosis of Alzheimer disease. Arch Neurol.

[31] Leung R (2013). Inflammatory proteins in plasma are associated with severity of Alzheimer’s disease. PLoS One.

[32] Tang MX (1996). Effect of oestrogen during menopause on risk and age at onset of Alzheimer’s disease. Lancet.

[33] Verdile G (2014). Associations between gonadotropins, testosterone and beta amyloid in men at risk of Alzheimer’s disease. Mol Psychiatry.

[34] Deac OM (2015). Tryptophan catabolism and vitamin B-6 status are affected by gender and lifestyle factors in healthy young adults. J Nutr.

[35] Canuso CM, Pandina G (2007). Gender and schizophrenia. Psychopharmacol Bull.

[36] McGinty F, Rose DP (1969). Influence of androgens upon tryptophan metabolism in man. Life Sci.

[37] Rose DP (1972). Aspects of tryptophan metabolism in health and disease: a review. J Clin Pathol.

[38] Rose DP (1967). The influence of sex, age and breast cancer on tryptophan metabolism. Clin Chim Acta.

[39] de Bie J, Lim CK, Guillemin GJ (2016). Kynurenines, Gender and Neuroinflammation; Showcase Schizophrenia. Neurotoxicity research.

[40] Neu SC (2017). Apolipoprotein E Genotype and Sex Risk Factors for Alzheimer Disease: A Meta-analysis. JAMA Neurol.

[41] Brann DW, Dhandapani K, Wakade C, Mahesh VB, Khan MM (2007). Neurotrophic and neuroprotective actions of estrogen: basic mechanisms and clinical implications. Steroids.

[42] Fillit H (1986). Observations in a preliminary open trial of estradiol therapy for senile dementia-Alzheimer’s type. Psychoneuroendocrinology.

[43] Ohkura T (1994). Evaluation of estrogen treatment in female patients with dementia of the Alzheimer type. Endocr J.

[44] Kawas C (1997). A prospective study of estrogen replacement therapy and the risk of developing Alzheimer’s disease: the Baltimore Longitudinal Study of Aging. Neurology.

[45] Bialek M, Zaremba P, Borowicz KK, Czuczwar SJ (2004). Neuroprotective role of testosterone in the nervous system. Pol J Pharmacol.

[46] Jones SP (2015). Expression of the Kynurenine Pathway in Human Peripheral Blood Mononuclear Cells: Implications for Inflammatory and Neurodegenerative Disease. PLoS One.

[47] Akimoto H, Yamada A, Takikawa O (2007). Up-regulation of the brain indoleamine 2,3-dioxygenase activity in a mouse model of Alzheimer’s disease by systemic endotoxin challenge. International Congress Series.

[48] Fukui S, Schwarcz R, Rapoport SI, Takada Y, Smith QR (1991). Blood-brain barrier transport of kynurenines: implications for brain synthesis and metabolism. J Neurochem.

[49] Lim CK (2017). Kynurenine pathway metabolomics predicts and provides mechanistic insight into multiple sclerosis progression. Sci Rep.

[50] Lynch MA (2014). The impact of neuroimmune changes on development of amyloid pathology; relevance to Alzheimer’s disease. Immunology.

[51] Suo Z (1998). Alzheimer’s beta-amyloid peptides induce inflammatory cascade in human vascular cells: the roles of cytokines and CD40. Brain Res.

[52] Werner-Felmayer G (1989). Characteristics of interferon induced tryptophan metabolism in human cells *in vitro*. Biochim Biophys Acta.

[53] Guillemin GJ, Smythe GA, Veas LA, Takikawa O, Brew BJ (2003). A beta 1-42 induces production of quinolinic acid by human macrophages and microglia. Neuroreport.

[54] Smith MA (2010). Increased iron and free radical generation in preclinical Alzheimer disease and mild cognitive impairment. J Alzheimers Dis.

[55] Chobot V, Hadacek F, Weckwerth W, Kubicova L (2015). Iron chelation and redox chemistry of anthranilic acid and 3-hydroxyanthranilic acid: A comparison of two structurally related kynurenine pathway metabolites to obtain improved insights into their potential role in neurological disease development. J Organomet Chem.

[56] Nasreddine ZS (2005). The Montreal Cognitive Assessment, MoCA: a brief screening tool for mild cognitive impairment. J Am Geriatr Soc.

[57] Rossetti HC, Lacritz LH, Cullum CM, Weiner MF (2011). Normative data for the Montreal Cognitive Assessment (MoCA) in a population-based sample. Neurology.

[58] McKhann GM (2011). The diagnosis of dementia due to Alzheimer’s disease: recommendations from the National Institute on Aging-Alzheimer’s Association workgroups on diagnostic guidelines for Alzheimer’s disease. Alzheimers Dement.

[59] Folstein MF, Folstein SE, McHugh PR (1975). “Mini-mental state”. A practical method for grading the cognitive state of patients for the clinician. J Psychiatr Res.

[60] Zhou L (2014). MR-less surface-based amyloid assessment based on 11C PiB PET. PLoS One.

[61] Bourgeat P (2015). Comparison of MR-less PiB SUVR quantification methods. Neurobiol Aging.

[62] Smythe GA (2002). Concurrent quantification of quinolinic, picolinic, and nicotinic acids using electron-capture negative-ion gas chromatography-mass spectrometry. Anal Biochem.

