# Supplementary material for: Alterations in serum kynurenine pathway metabolites in individuals with high neocortical amyloid-β load: A pilot study
Source: Sci Rep. 2018 May 22;8:8008. doi: 10.1038/s41598-018-25968-7 (PMC5964182; doi:10.1038/s41598-018-25968-7)
Supplement: Supplementary file 1 — Supplementary Information [file 41598_2018_25968_MOESM1_ESM.docx]

**Alterations in serum kynurenine pathway metabolites in individuals with high neocortical amyloid-β load: A pilot study**

Pratishtha Chatterjee, Ph.D. ^ǂ,a,b^, Kathryn Goozee, MCN ^ǂ,a,b,c,d,e,f,g,^, Chai K. Lim, Ph.D. ^ǂ,a^, Ian James, Ph.D. ^h^, Kaikai Shen, Ph.D. ^i^, Kelly R. Jacob, Ph.D. ^a^, Hamid R. Sohrabi, Ph.D. ^a,b,e,f^, Tejal Shah, Ph.D. ^a,b,f^, Prita R. Asih, B.Sc. ^c,j^ , Preeti Dave, M.Sc. ^a,d^, Candice ManYan, B.Tech ^d^, Kevin Taddei, B.Sc. ^b,f^, David B. Lovejoy, Ph.D. ^a^, Roger Chung, Ph.D. ^a^, Gilles J. Guillemin, Ph.D. ^a^, Ralph N. Martins, Ph.D.*^,a,b,c,e,f,g^

**Supplementary material**

**Supplementary Table 1. Demographic characteristics of female and male cohort participants, separately.** Baseline characteristics in the female and male cohort subsets including age, body mass index (BMI), *APOE ε4* status, mini mental state examination (MMSE) scores, Montreal Cognitive Assessment (MoCA, adjusted for education) scores, years of education, neocortical amyloid load (NAL) represented by the standard uptake value ratio (SUVR) of ligand ^18^F-Florbetaben (FBB) in the neocortical region normalised with that in the cerebellum and hippocampal volume (HV) normalised by the intracranial volume, have been compared between NAL- (SUVR<1.35) and NAL+ (SUVR≥1.35) study participants. Chi-square test or linear models were employed as appropriate. Sixty-five female participants (nNAL- = 45, nNAL+ = 20) and 31 male participants (nNAL- = 19, nNAL+ = 12) underwent MRI.

|  | **Females** | | | **Males** | | |
| --- | --- | --- | --- | --- | --- | --- |
|  | **NAL-** | **NAL+** | **p** | **NAL-** | **NAL+** | **p** |
| N | 46 | 22 | - | 19 | 13 | - |
| Age (years, mean ±SD) | 77.02 ± 5.09 | 78.18 ± 5.60 | .398 | 79.05 ± 6.45 | 81.00 ± 4.65 | .359 |
| BMI (mean ±SD) | 27.48 ± 4.91 | 28.12 ± 5.62 | .632 | 27.13 ± 3.29 | 27.92 ± 2.84 | .488 |
| n*APOE ε4* carriers (%) | 5 (10.8) | 12 (54.5) | **.0001** | 0 (0) | 4 (30.7) | **.010** |
| MMSE (mean ±SD) | 28.65 ± 1.05 | 28.90 ± 1.19 | .372 | 28.15 ± 1.34 | 28.61 ± .96 | .300 |
| MoCA (mean ±SD) | 27.59 ± 1.61 | 27.09 ± 1.90 | .267 | 27.05 ± 1.78 | 26.92 ± 2.02 | .849 |
| Education (years, mean ±SD) | 13.67 ± 2.53 | 12.84 ± 2.94 | .234 | 17.68 ± 3.51 | 15.00 ± 2.41 | **.023** |
| FBB-PET SUVR (mean ±SD, nF=68, nM=32) | 1.14 ± 0.08 | 1.71 ± 0.26 | - | 1.19 ± .08 | 1.70 ± .26 | - |
| HV% (left; right lobes, mean ±SD, nF=65, nM=31) | 0.197 ± 0.019;  0.202 ± 0.018 | 0.198 ± 0.019;  0.201 ± 0.017 | .829,  .904 | 0.190 ± .022;  0.192 ± .025 | 0.187 ± .018;  0.196 ± .021 | .671, .597 |

*#On comparing SUVRs of males (mean±SD: 1.40±.31) vs females (mean±SD: 1.33±.32), adjusting for age and APOE ε4 carriage, no significant difference was observed (p=.155). Further, within NAL- participants, no significant difference was observed between SUVRs of males (mean±SD: 1.19±.085) vs females (mean±SD: 1.14±.083), adjusting for age and APOE ε4 carriage (p=.067). Similarly, within NAL+ participants, no significant difference was observed between SUVRs of males (mean±SD: 1.71±.27) vs females (mean±SD: 1.72±.26), adjusting for age and APOE ε4 carriage (p=.896).*

**Supplementary Table 2.** **Correlation of kynurenine pathway metabolites with age in all participants.** Correlation between kynurenine pathway metabolites and age using Pearson’s correlation coefficient (r) and partial correlation coefficient r^a^, adjusting for covariates gender and *APOE* ε4 status and r^b^ adjusting for gender, *APOE* ε4 status and years of education, have been represented for all study participants (n=100). All p values were obtained from variables transformed to the logarithmic scale for analyses to meet assumptions of the statistical test employed. p<.05 was considered significant, NS represents non-significant p-values, p>.1.

|  | **r** | **p** | **r^a^** | **p^a^** | **r^b^** | **p^b^** |
| --- | --- | --- | --- | --- | --- | --- |
| Tryptophan (TRP, µM) | -0.180 | 0.073 | -.189 | .062 | -.180 | .078 |
| Kynurenine (KYN, µM) | 0.169 | 0.093 | .169 | .095 | .189 | .063 |
| Kynurenic Acid (KYNA, nM) | .202 | **0.044** | .140 | .169 | .151 | .141 |
| 3-hydroxykynurenine (3-HK, nM) | 0.161 | 0.109 | .138 | .176 | .154 | .131 |
| 3-hydroxyanthranilic acid (3-HAA, nM) | -0.092 | 0.363 | -.186 | .067 | -.172 | .091 |
| Anthranilic acid (AA, nM) | .196 | 0.050 | .169 | .097 | .185 | .070 |
| Picolinic acid (nM) | 0.121 | 0.232 | .109 | .287 | .157 | .125 |
| Quinolinic acid (nM) | .239 | **0.017** | .237 | **.019** | .253 | **.012** |

**Supplementary Table 3.** **Comparison of kynurenine pathway metabolites between *APOE* ε4 non-carriers and carriers.** Kynurenine pathway metabolites measured in the serum were compared between *APOE* ε4 non-carrier and carrier study participants, employing linear models. p<.05 was considered as significant. p^a^ indicates p values adjusted for covariates age and gender and p^b^ indicates p values adjusted for covariates age, gender and years of education. All p values were obtained from variables transformed to the logarithmic scale for analyses to meet assumptions of the statistical test employed.

|  | **Non-carriers**  **(n=79)** | | **Carriers**  **(n=21)** | |  |  |  |
| --- | --- | --- | --- | --- | --- | --- | --- |
|  | **Mean** | **SD** | **Mean** | **SD** | **p** | **p^a^** | **p^b^** |
| Tryptophan (µM) | 42.99 | 7.80 | 44.61 | 7.44 | .365 | .401 | .375 |
| Kynurenine (KYN, µM) | 2.20 | .57 | 2.30 | .60 | .442 | .317 | .243 |
| Kynurenic Acid (KYNA, nM) | 53.17 | 29.92 | 47.89 | 14.51 | .787 | .694 | .626 |
| 3-hydroxykynurenine (3-HK, nM) | 121.79 | 40.53 | 110.60 | 20.88 | .271 | .390 | .480 |
| 3-hydroxyanthranilic acid (3-HAA, nM) | 22.89 | 9.20 | 21.61 | 9.03 | .625 | .904 | .978 |
| Anthranilic acid (AA, nM) | 34.98 | 19.11 | 37.33 | 22.56 | .562 | .296 | .236 |
| Picolinic acid (nM) | 111.64 | 53.43 | 100.87 | 32.44 | .371 | .452 | .692 |
| Quinolinic acid (nM) | 172.48 | 77.92 | 169.37 | 61.37 | .956 | .747 | .635 |

**Supplementary Table 4.** **Comparison of kynurenine pathway metabolites between males and females.** Kynurenine pathway metabolites measured in the serum were compared between male and female study participants, employing linear models. p<.05 was considered as significant. p^a^ indicates p values adjusted for covariates age and *APOE* ε4 status and p^b^ indicates p values adjusted for covariates age, *APOE* ε4 status and years of education. All p values were obtained from variables transformed to the logarithmic scale for analyses to meet assumptions of the statistical test employed.

|  | **Males**  **(n=32)** | | **Females**  **(n=68)** | |  |  |  |
| --- | --- | --- | --- | --- | --- | --- | --- |
|  | **mean** | **SD** | **mean** | **SD** | **p** | **p^a^** | **p^b^** |
| Tryptophan (µM) | 43.90 | 7.61 | 43.06 | 7.81 | .604 | .310 | .484 |
| Kynurenine (KYN, µM) | 2.25 | .49 | 2.21 | .61 | .567 | .723 | .782 |
| Kynurenic Acid (KYNA, nM) | 66.74 | 36.75 | 45.15 | 18.32 | **.0001** | **.0004** | **.005** |
| 3-hydroxykynurenine (3-HK, nM) | 122.34 | 28.94 | 118.08 | 40.99 | .336 | .592 | .985 |
| 3-hydroxyanthranilic acid (3-HAA, nM) | 27.74 | 9.92 | 20.21 | 7.71 | **.0001** | **.00006** | **.001** |
| Anthranilic acid (AA, nM) | 41.65 | 24.00 | 32.57 | 16.88 | **.036** | .063 | .250 |
| Picolinic acid (nM) | 109.35 | 37.43 | 109.39 | 54.94 | .696 | .959 | .226 |
| Quinolinic acid (nM) | 177.52 | 81.26 | 169.15 | 71.54 | .651 | 1 | .601 |
| Anthranilic acid to kynurenic acid ratio | 9.95 | 4.94 | 8.70 | 4.47 | .191 | .267 | .593 |

**Supplementary Table 5. Comparison of serum** **kynurenine pathway metabolites between NAL- and NAL+ participants.** Kynurenine pathway metabolites measured in the serum were compared between participants with neocortical amyloid-β load (NAL, assessed by the standard uptake value ratio observed via positron emission tomography using ligand ^18^F-florbetaben) <1.35 (NAL-) and ≥1.35 (NAL+) employing linear models. Kynurenine to tryptophan ratios for all participants were multiplied by 1000. p<.05 was considered as significant. p^a^ indicates p values adjusted for covariates age, gender and *APOE* ε4 status while p^b^ indicates p values adjusted for covariates age, gender, *APOE* ε4 status and years of education. All p values were obtained from variables transformed to the logarithmic scale for analyses to meet assumptions of the statistical test employed.

| KP metabolite | NAL-  (mean±SD) | NAL+  (mean±SD) | p | p^a^ | p^b^ |
| --- | --- | --- | --- | --- | --- |
| Tryptophan (µM) | 42.87±8.15 | 44.17±6.87 | .350 | .325 | .341 |
| Kynurenine (KYN, µM) | 2.12±0.52 | 2.41±0.63 | **.017** | .051 | **.028** |
| Kynurenic Acid (KYNA, nM) | 49.71±26.25 | 56.41±29.40 | .238 | .287 | .412 |
| 3-hydroxykynurenine (3-HK, nM) | 117.74±34.73 | 122.60±42.42 | .554 | .357 | .284 |
| 3-hydroxyanthranilic acid (3-HAA, nM) | 22.15±9.25 | 23.50±8.99 | .406 | .159 | .255 |
| Anthranilic acid (AA, nM) | 31.40±14.99 | 43.04±25.02 | **.003** | **.008** | **.006** |
| Picolinic acid (nM) | 112.06±56.25 | 104.40±35.07 | .572 | .667 | .964 |
| Quinolinic acid (nM) | 169.76±75.89 | 175.66±72.71 | .689 | .927 | .908 |
| Kynurenine to tryptophan ratio (K:T) | 50.73±13.94 | 56.08±17.58 | .121 | .248 | .165 |
| Anthranilic acid to kynurenic acid ratio | 8.14±3.50 | 10.89±5.87 | **.003** | **.009** | **.006** |

**Supplementary Table 6. Comparison of kynurenine pathway metabolites between NAL- and NAL+, in males and females.** Kynurenine pathway metabolites measured in the serum were compared separately in males and females, between participants with neocortical amyloid-β load (NAL, assessed by the standard uptake value ratio observed via positron emission tomography using ligand ^18^F-florbetaben) <1.35 (NAL-) and ≥1.35 (NAL+) employing linear models. p<.05 was considered as significant. p^a^ indicates p values adjusted for covariates age and *APOE* ε4 status while p^b^ indicates p values adjusted for covariates age, *APOE* ε4 status and years of education. Kynurenine to tryptophan ratios for all participants were multiplied by 1000. All p values were obtained from variables transformed to the logarithmic scale for analyses to meet assumptions of the statistical test employed.

|  | **Males (n=32)** | | | | | | | **Females (n=68)** | | | | | | |
| --- | --- | --- | --- | --- | --- | --- | --- | --- | --- | --- | --- | --- | --- | --- |
|  | NAL- (n=19) | | NAL+ (n=13) | |  | | | NAL- (n=46) | | NAL+ (n=22) | |  | | |
|  | Mean | SD | Mean | SD | p | p^a^ | p^b^ | Mean | SD | Mean | SD | p | p^a^ | p^b^ |
| Tryptophan (µM) | 43.62 | 8.08 | 44.31 | 7.159 | .760 | .931 | .751 | 42.57 | 8.25 | 44.08 | 6.87 | .382 | .365 | .368 |
| Kynurenine (KYN, µM) | 2.33 | .44 | 2.14 | .55 | .207 | .451 | .870 | 2.03 | .53 | 2.58 | .63 | **.0004** | **.004** | **.003** |
| Kynurenic Acid (KYNA, nM) | 68.86 | 37.38 | 63.66 | 37.10 | .551 | .864 | .767 | 41.81 | 14.24 | 52.13 | 23.68 | .068 | .207 | .207 |
| 3-hydroxykynurenine (3-HK, nM) | 132.79 | 30.58 | 107.06 | 18.30 | **.008** | **.022** | **.017** | 111.53 | 34.74 | 131.78 | 49.84 | **.037** | **.017** | **.014** |
| 3-hydroxyanthranilic acid (3-HAA, nM) | 27.95 | 10.91 | 27.43 | 8.69 | .921 | .893 | .431 | 19.75 | 7.34 | 21.18 | 8.51 | .523 | .301 | .317 |
| Anthranilic acid (AA, nM) | 40.51 | 18.91 | 43.32 | 30.76 | .947 | .691 | .783 | 27.64 | 11.27 | 42.87 | 21.74 | **.0001** | **.001** | **.001** |
| Picolinic acid (nM) | 118.01 | 40.55 | 96.69 | 29.34 | .083 | .139 | .525 | 109.60 | 61.82 | 108.96 | 37.96 | .744 | .674 | .600 |
| Quinolinic acid (nM) | 194.40 | 80.73 | 152.84 | 78.55 | .068 | .109 | .213 | 159.58 | 72.26 | 189.15 | 67.24 | .067 | .247 | .229 |
| Kynurenine to tryptophan ratio (K:T) | 55.18 | 14.63 | 49.53 | 16.10 | .230 | .469 | .710 | 48.89 | 13.38 | 59.95 | 17.61 | **.006** | **.033** | **.031** |
| Anthranilic acid to kynurenic acid ratio | 9.75 | 4.46 | 10.24 | 5.74 | .817 | .628 | .809 | 7.47 | 2.82 | 11.27 | 6.05 | **.0004** | **.003** | **.001** |

**Supplementary Table 7.** **Correlation between kynurenine pathway metabolites and NAL, in males and females.** Correlation between kynurenine pathway metabolites and neocortical amyloid-β load (NAL) using Pearson’s correlation coefficient (r) and partial correlation coefficient r^a^ adjusting for covariates age and *APOE* ε4 status and r^b^ adjusting for covariates age, *APOE* ε4 status and years of education, have been represented separately in males and females. All p values were obtained from variables transformed to the logarithmic scale for analyses to meet assumptions of the statistical test employed. p<.05 was considered significant, NS represents non-significant p values, p>.1.

|  | **Males (n=32)** | | | | | | **Females (n=68)** | | | | | |
| --- | --- | --- | --- | --- | --- | --- | --- | --- | --- | --- | --- | --- |
|  | **r** | **p** | **r^a^** | **p^a^** | **r^b^** | **p^b^** | **r** | **p** | **r^a^** | **p^a^** | **r^b^** | **p^b^** |
| Tryptophan (µM) | .095 | NS | .097 | NS | .146 | NS | .058 | NS | .062 | NS | .062 | NS |
| Kynurenine (KYN, µM) | -.059 | NS | .049 | NS | .178 | NS | .346 | **.004** | .259 | **.036** | .261 | **.036** |
| Kynurenic Acid (KYNA, nM) | -.050 | NS | .013 | NS | .103 | NS | .161 | NS | .086 | NS | .086 | NS |
| 3-hydroxykynurenine (3-HK, nM) | -.161 | NS | -.064 | NS | -.055 | NS | .232 | .057 | .255 | **.039** | .259 | **.037** |
| 3-hydroxyanthranilic acid (3-HAA, nM) | -.045 | NS | -.018 | NS | .095 | NS | .222 | .069 | .299 | **.015** | .299 | **.016** |
| Anthranilic acid (AA, nM) | .099 | NS | .162 | NS | .148 | NS | .449 | **.0001** | .382 | **.002** | .398 | **.001** |
| Picolinic acid (nM) | -.241 | NS | -.207 | NS | -.049 | NS | .026 | NS | .028 | NS | .031 | NS |
| Quinolinic acid (nM) | -.165 | NS | -.120 | NS | -.045 | NS | .222 | .069 | .136 | NS | .138 | NS |
